# Control and Prevention of SARS-CoV-2 Outbreaks among Healthcare Workers from 129 Healthcare Facilities in Mexico

**DOI:** 10.3390/ijerph182211772

**Published:** 2021-11-10

**Authors:** César Pineda-Santoyo, Abraham Campos-Romero, Marco A. Luna-Ruiz Esparza, Liliana E. López-Luna, Martha E. Sánchez-Zarate, Abraham Zepeda-González, Miguel A. Fernández-Rojas, Jonathan Alcántar-Fernández

**Affiliations:** 1 Hygiene and Occupational Safety Department, Salud Digna, Culiacan 80000, Mexico; cesar.pineda@salud-digna.org; 2 Human Resources Department, Salud Digna, Culiacan 80000, Mexico; 3 Innovation and Research Department, Salud Digna, Culiacan 80000, Mexico; abraham.campos@salud-digna.org (A.C.-R.); marco.luna@salud-digna.org (M.A.L.-R.E.); miguel.fernandez@salud-digna.org (M.A.F.-R.); 4 Tijuana Centro Clinic, Salud Digna, Tijuana 22000, Mexico; liliana.lopez@salud-digna.org; 5 Gustavo A. Madero Clinic, Salud Digna, Ciudad de Mexico 07020, Mexico; martha.sanchez@salud-digna.org; 6 Institutional Relations Department, Salud Digna, Culiacan 80000, Mexico; abraham.zepeda@salud-digna.org

**Keywords:** healthcare workers, SARS-CoV-2, COVID-19, occupational health program, workflow

## Abstract

Few reports have shared the workflows to reduce SARS-CoV-2 infections among risk groups, including healthcare workers (HCWs). This study describes an occupational health program implemented to reduce the incidence of COVID-19 and establishes a back-to-work algorithm in HCWs of 129 Salud Digna outpatient care clinics in Mexico. This program was composed of training plans, screening SARS-CoV-2 infections, the containment of infections, follow-up COVID-19 cases, and continuing supervision in addition to the steady supply and training for the correct use of PPE. From 16 April 2020 to 15 April 2021, 7376 individuals were enrolled, of which 423 were excluded because they did not meet the inclusion criteria or refused the follow-up. In the cohort studied, we found a COVID-19 incidence of 35.4% (2610 individuals), lower hospitalization (0.11%), ICU (0.04%) and lethality rate (0.04%). Additionally, 85.9% of COVID-19 cases tested negative for SARS-CoV-2 after 14 days of the first positive test with an average isolation time of 26–33 days. Finally, 99% of people received personal protective equipment and adequate training to use it. Our results show that the program implemented reduced the hospitalization ICU admission and lethality in HCWs; we consider this workflow to help other workplaces offer safe conditions for HCWs and patients.

## 1. Introduction

Around the world, healthcare workers (HCWs) are essential to the function of health systems; during the SARS-CoV-2 pandemic, they have cared for people who are ill to prevent the progression of severe disease and death. One of the goals of governments is to avoid the collapse of healthcare systems. Thus, different strategies need to be implemented to protect HCWs, who, compared to the general population, are occupationally exposed to SARS-CoV-2 infections [1]. Previous reports have shown SARS-CoV-2 infections to range from 7 to 11% among HCWs [1,2]. In this regard, Mexico is a country with a high incidence and mortality rate of COVID-19 among HCWs, reporting 182,246 cases and 4127 COVID-19 related deaths from 27 February 2020 to 2 August 2021 [3,4,5].

An incidence of 30.35% of infections in HCWs with a lethality rate of 0.82% in Mexico City HCWs has been previously reported [6]. In addition, most COVID-19 cases are ambulatory, thus highlighting the urgency to make interventions to reduce the SARS-CoV-2 spread among HCWs since they constitute the basis of the health system [7].

Additionally, the higher risk of SARS-CoV-2 infection in HCWs is due to their closer contact with infected individuals or non-confirmed asymptomatic cases; other factors such as long workdays, shortage of personal protective equipment (PPE), and inadequate training in PPE usage, have been identified as the leading causes of infection for HCWs [8,9,10].

Moreover, implementing actions, such as the early recognition of suspected COVID-19 cases and strict hygiene practices, has shown benefits in reducing SARS-CoV-2 infections and COVID-19 related mortality in healthcare facilities [11,12]. However, few reports have shared the policies and programs implemented to mitigate the COVID-19 cases in their facilities.

Therefore, the present study aims to share the workflow and results of implementing an occupational health program (COVID-19 control program) designed to reduce and control SARS-CoV-2 infections in 129 outpatient primary care facilities around Mexico.

## 2. Materials and Methods

### 2.1. Study Design and Population Cohort

A multicenter, prospective cohort study (16 April 2020 to 15 April 2021) of healthcare workers (HCWs) during the COVID-19 pandemic was carried out. We recruited HCWs of any sex and adults under 60 years of age in 129 Salud Digna primary-care clinics located in 32 states of Mexico. We analyzed clinical, occupational, and sociodemographic information, training provided, and PPE use.

We classified HCWs according to their jobs as front-line COVID-19 (front-line HCW) or non-front-line COVID-19 (non-front-line HCW). Frontline HCW jobs include physicians, medical assistants, radiologists, radiologic technicians, medical lab technologists, and molecular biologists (collecting, receiving, or processing samples for COVID-19 diagnosis). Non-front-line HCW jobs entail administrative staff, nutritionists, optometrists, staff from supporting areas (IT engineers, biomedical engineers, facility maintenance, and security), and nurses who did not directly attend to COVID-19 patients.

### 2.2. Inclusion and Exclusion Criteria

We enrolled individuals testing positive for SARS-CoV-2 infection, detected by quantitative real-time PCR (rRT-PCR) or antigen-detecting rapid diagnostic test (ag-RDT). Ag-RDT were used in facilities located in states of the country where there were no available rRT-PCR tests, according to WHO guidance [13]. From people who consented to the follow-up, we collect demographic, clinical, PPE use, and COVID-19 training program information for research purposes.

Moreover, as part of the occupational health program and in agreement with Mexican Ministry of Health policies, we identified those workers with health conditions of potential risk for severe COVID-19 illness, such as diabetes, obesity, hypertension, cancer, lupus, AIDS, or arthritis, aged ≥60 years old, as well as pregnant women [14,15,16,17]. Therefore, they were isolated in their homes; consequently, they were excluded from this report.

### 2.3. Molecular and Immunological SARS-CoV-2 Test

We detected the SARS-CoV-2 by real-time reverse transcription-polymerase chain reaction (rRT-PCR) in the molecular biology laboratories at the National Reference Centers located in Mexico State and Culiacan, Sinaloa [18]. Briefly, we extracted viral RNA from nasopharyngeal swabs in an automated manner. Additionally, we used the automated Cobas 6800 (Roche Diagnostic, Tucson, AZ, USA) and QuantStudio 7 Flex (Thermo Fisher, San Francisco, CA, USA) for PCR tests following the manufacturer’s instructions. For SARS-CoV-2 detection, we used the cobas^®^ SARS-CoV-2 Test (Roche Diagnostics, Tucson, AZ, USA), TaqMan 2019-nCoV Assay Kit v1 (Thermo Scientific, San Francisco, CA, USA), and VIASURE SARS-CoV-2 Real-Time PCR Detection Kit (CerTest Biotec, San Mateo de Gállego, Zaragoza, Spain) that previously has shown no significant bias [18]. Positive results were interpreted as target gene amplification under cycle 38 (Ct < 38) [18].

Furthermore, in settings where there was no PCR test available, we used the SARS-CoV-2 Rapid Antigen Test (BIOSENSOR, Roche Diagnostics, Tucson, Arizona, US). Briefly, we collected nasopharyngeal swabs, took 350 µL of the viral transport medium, and mixed it with the extraction buffer provided in the kit; then, we placed 3 drops of the mixture in the nitrocellulose test strip and incubated it for 15–30 min at room temperature. According to the manufacturer’s instructions, positive results were interpreted as the presence of any line, regardless of its thickness, in the region covered with anti-mouse-SARS-CoV-2 monoclonal antibody (T). This test is reported to have good clinical performance [19] and the Emergency Use Authorization (EUA) by the Ministry of Health in the country [20]. Laboratories have ISO 9001 certification granted by the Mexican Institute of Normalization and Certification, with a technical capacity endorsement by the National Institute of Epidemiological Diagnosis and Reference (InDRE) from the Ministry of Health.

### 2.4. COVID-19 Control Program

On 27 February 2020, the first case of COVID-19 in Mexico was detected [21]; following, we designed this program to manage COVID-19 incidence and reduce the lethality by COVID-19. On 17 March 2020, we implemented a COVID-19 control program composed of a set of hygiene policies and training programs applied in 129 ambulatory Salud Digna clinics located in 32 states of Mexico. This program focused on screening positive cases and implementing mitigation actions to interrupt the contagion chains, reducing the spread of the SARS-CoV-2 virus and new infections through implementing 5 phases in parallel: (1) training to prevent SARS-CoV-2 spread and disinfection protocols, (2) screening and triage, (3) containment actions and (4) follow-up positive cases, and (5) supervision and monitoring.

Phase 1: Training to prevent SARS-CoV-2 spread

We performed remote and on-site training programs, including handwashing, surface disinfection, and adequate PPE use workshops. Additionally, we included training to identify COVID-19-related symptoms and contingency actions to prevent SARS-CoV-2 spread. We used different ways to share information to reinforce training, such as the institutional training platform, videos, slide presentations, emails, and the institutional bulletin.

Phase 2: Screening and triage

1. Triage in facilities

We implemented a triage for all HCWs at the clinics’ entry that included: measuring body temperature, looking for fever (T ≥ 38 °C), and asking for COVID-19 related symptoms such as fever, cough, body pain, chills, anosmia, and ageusia. Symptomatic people were tagged as COVID-19 suspicious, and were home isolated and referred to medical staff for further evaluation. Moreover, we provided N95 face masks and hand sanitizing gel for people who were allowed to work. Furthermore, we continuously supervised them to ensure they were using PPE correctly during work (Supplementary Figure S1).

2. Screening in facilities

We randomly tested front-line healthcare workers for SARS-CoV-2 by qRT-PCR every week, evaluating different individuals each time to ensure they were all tested. Furthermore, we evaluated workers from clinics with at least five positive COVID-19 cases in the previous two weeks. Those who made work-related trips were tested before and after the trip.

3. SARS-CoV-2 test scheduling

All workers who self-reported COVID-19 related symptoms or were tagged as COVID-19 suspicious in the triage were interviewed by phone call or video call by the medical staff. We used the EpiSD app to collect information during the interview, which utilized a standardized COVID-19 risk questionnaire (Supplementary Table S1) developed for triage of suspected COVID-19 cases. On this basis, people: (a) returned to activities, (b) were referred for medical assistance, (c) were recommended isolation for 2–7 days, or (d) were scheduled for SARS-CoV-2 PCR test or rapid antigen test (Supplementary Figure S1).

Phase 3: Mitigation actions

1. Identification and isolation of positive cases

Individuals suspected of COVID-19 with negative PCR tests were isolated for five days. Then, their back-to-work was evaluated and approved by the medical staff. Moreover, COVID-19 confirmed cases by PCR or rapid antigen test were isolated for at least 14 or 21 days, respectively, since the date of onset symptoms and were followed-up by medical staff (Supplementary Figure S1) according to the workflow of the COVID-19 program described in the following sections.

2. Epidemiologic study

We interviewed people diagnosed with COVID-19 to conduct epidemiologic studies using a standardized survey based on the Epidemiological Study of Suspicious Case of Viral Respiratory Disease, published by Mexico’s Ministry of Health [22] with slight modifications. We included mobility information, contacts studied, training, and PPE use (Supplementary Table S2).

We classified the origin of contagion as community infections (the source was from outside Salud Digna facilities) or internal infection (when the contagion originated inside facilities by co-workers or by patients). When an outbreak was detected, we reinforced training, screening, and disinfection protocols.

3. Disinfection of surfaces and facilities

We implemented continuous disinfection with microdacyn^®^ in waiting rooms and each consultant room before attending to each patient. At the end of the workday, facilities were disinfected using chlorine solutions. In contrast, we applied specific measures such as sanitization of the entire work center and strengthening staff training in clinics with at least 5 confirmed COVID-19 cases in two weeks.

Phase 4: Follow-up positive cases

The COVID-19 medical group of Salud Digna, integrated by 21 physicians specialized in occupational health and pharmacology, began the follow-up by phone calls of confirmed cases every third day until their recovery. In addition, one nutritionist and three engineers supported the physicians to ensure the implementation of all phases of the occupational program herein described. Consequently, this allowed the identification of health status of patients and to provide tailored recommendations according to their characteristics, in some cases we provided prescription of medications (e.g., pain relievers, anti-inflammatory, antipyretics, and cough suppressants), recommendations to carry out lab tests (e.g., D-dimer, ferritin, C-reactive protein, hemogram) or tomography, suggestions to see a general practitioner or specialist for a physical evaluation or even go to a hospital, if this was required. These actions helped us to make timely decisions to refer HCWs for medical attention to avoid possible complications.

Followed up cases were classified into six groups according to symptoms and severity as follows:

1. Mild case: people who were asymptomatic or presented symptoms that were tolerable, temporary, and did not put their lives at risk, such as headache, fatigue, myalgia, anosmia, ageusia, runny nose, or sore throat.
2. Moderate case: people who received ambulatory assisted oxygenation having difficulty breathing, standing difficulty, and excessive cough that did not allow exertion.
3. Hospitalized case: people who presented symptoms as a moderate case and needed specialized assistance in public or private health institutions.
4. Respiratory support case: people who needed high flow oxygen due to respiratory insufficiency.
5. ICU case: people who needed mechanical ventilation and intensive care due to severe respiratory insufficiency.
6. Death case: people died due to COVID-19.

### 2.5. Criteria for Discharge HCW from Follow-Up and Back to Work

People who were followed up were tested for SARS-CoV-2 by q-RT-PCR every 14 days; if the result was negative, they were discharged and returned to the workplace only if they had no symptoms for at least 5 days prior to the PCR test or they had symptoms similar to mild cases. If the PCR test was positive and continued having symptoms, people were referred for specialized medical assessment, continued isolation with follow-up until they were cleared of any symptoms. They were tested again by PCR to define their return to work.

If a PCR positive result persisted (SARS-CoV-2 positive persistent), we evaluated the presence of symptoms as a discriminant parameter. For example, people were discharged considering the absence of symptoms in the last five days, the presence of symptoms similar to a mild case, or if their positive results had a Ct value ≥38 (Supplementary Figure S2).

Phase 5: Supervision and monitoring

We established a committee that supervised this workflow, meeting twice a week, evaluating the incidence of COVID-19 in all clinics, and approving deep sanitization in facilities. Furthermore, the committee continuously monitored the adherence to internal policies to prevent outbreaks, the training provided, and PPE use. Furthermore, we analyzed the epidemiologic case studies to create dashboards to daily monitor several indicators, such as contagion origin, COVID-19 incidence by clinic and state, and the status of people with COVID-19 (Supplementary Figure S3). The above helps to make specific decisions for each clinic and better manage the impact of the pandemic in healthcare facilities.

### 2.6. Statistical Analysis

We used SPSS 23 (SPSS Inc., Chicago, IL, USA) and GraphPad Prism 8 (GraphPad Software Inc., San Diego, CA, USA) for data analysis and graphics, considering a *p* ≤ 0.05 value as a statistically significant threshold in all tests. Categorical variables are shown as frequencies, while continuous variables are expressed as measures of central tendency. SARS-CoV-2 infection frequency and the contagious place were compared between front-line HCW and non-front-line HCW by chi-squared test. We make dashboards to analyze epidemiologic studies and monitor the follow-up COVID-19 cases with Power BI^®^ (Microsoft, Redmond, WA, USA).

## 3. Results

### 3.1. Enrollment of Healthcare Workers in the Study

**Figure 1:**
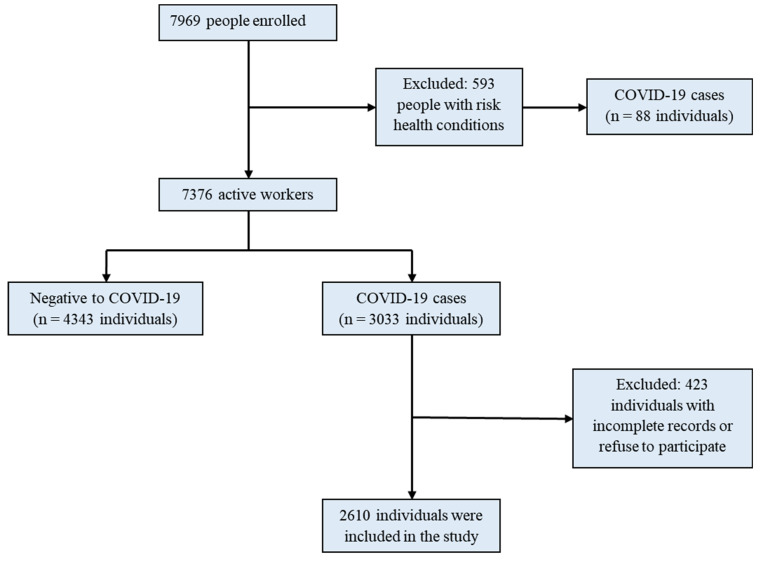
Flow chart of healthcare workers’ enrollment process to the study.

From 16 April 2020 to 15 April 2021, we enrolled 7969 individuals, of which 593 people were isolated in their homes due to their clinical risk conditions for severe COVID-19 illness. Then, 7376 people were identified as active workers, of which 4000 individuals (54.2% of the target population) were tested for SARS-CoV-2 infections by qRT-PCR. We identified 3033 positive individuals with PCR tests; however, we excluded 423 individuals due to not meeting the inclusion criteria or refusing the follow-up; then, we included 2610 individuals with COVID-19 in this study (Figure 1).

### 3.2. COVID-19 Incidence and Contagions Origin

**Figure 2:**
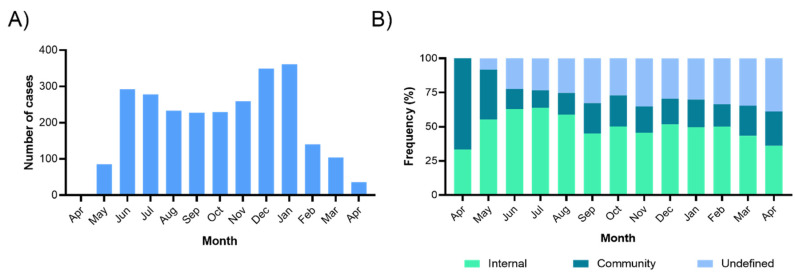
SARS-CoV-2 infection incidence during the first year of the COVID-19 control program implemented in Salud Digna clinics. (**A**) Monthly SARS-CoV-2 incidence in Salud Digna clinics from April 2020 to April 2021; (**B**) Monthly distribution of SARS-CoV-2 incidence by place of contagion: undefined, community (outside clinics), and internal (inside Salud Digna clinics).

Prior to the beginning of this program, the COVID-19 incidence was 1.8%. SARS-CoV-2 infections among active workers were 35.4%, with an average monthly incidence of 2.6%. In contrast, in workers with home isolation (due to health conditions), the incidence was 14.8%. Additionally, we observed an incidence decrease from May 2020 (85 cases) to April 2021 (36 cases) (Figure 2A). Regarding the origin of contagion, we observed that at the beginning of the pandemic, community infections were more frequent (70%) and then tended to decrease (11.5%) in the following months (May 2020 to April 2021), while internal infections (by co-workers and patients) increased in the first months with the maximum (60%) during the first epidemic curve (June to July 2020) and then decreased (30.0%); as the pandemic grew, the origin of contagion was more difficult to define (Figure 2B).

Our population studied was biased because more women (74.56%) than men (25.44%) were recruited; however, there were not significant differences (*p* = 0.0983) in infection rates between women (35.9 per 100 women, 95% CI = 34.7–37.2) and men (33.8 per 100 men, 95% CI = 31.8–36.0). Infections were more frequent among people younger than 30 years (44.2%), which tended to decrease by age (χ^2^ = 254.7, *p* < 0.0001). Moreover, infections were more frequent in front-line HCWs (56.7%) than non-front-line HCWs (43.2%); front-line HCWs had a higher infection rate (65.8 per 100 HCW, 95% CI = 63.8–67.7) than non-front-line HCWs (22.0 per 100 HCW, 95% CI = 20.9–23.2); this difference was statistically significant (*p <* 0.0001) (Table 1). As expected, infections in the workplace were more common in front-line HCWs (59.1%, 95% CI 56.6–61.6) than in non-front-line HCW (46.4%, 95% CI = 43.6–49.4) (*p <* 0.0001), while community infections were most among non-front-line HCWs (21.3%, 95% CI = 19.0–23.8) than front-line HCW (16.0%, 95% CI = 14.3–18.0) (*p <* 0.0001).

### 3.3. Baseline Characteristics of the Followed-Up Cohort

The medical group followed up HCWs diagnosed with COVID-19; of 2610 patients analyzed, the median age was 28 years (IQR 25–33), and 74.6% were females. The majority of COVID-19 cases (89.9%) were symptomatic at the time of PCR test, and 10.1% were presymptomatic (Table 2); the most frequent symptoms were headache (74.6%), myalgia (63.8%), sore throat (50.8%), and fever (49.4%). Moreover, 15.7% of people had at least one comorbidity, asthma (16.6%) and hyper/hypothyroidism (11.2%), being the common. Additionally, the majority of HCWs were non-smokers (88.5%) and used public transport (65.4%), which exposed them to more potential infections (Table 2).

### 3.4. Training Plan and Use of Personal Protective Equipment (PPE)

A training plan was continuously delivered that included identifying COVID-19 related symptoms, hand washing, and others. The majority of HCWs (99.9%) fulfilled the training plan, which included hand washing (93.9%), workplace disinfection (94.4%), and the use of masks (85.8%), among others. We delivered training programs mainly through the internal digital platform Universidad Salud Digna (55.2%), video recording (51.7%), and video call (41.9%). Furthermore, we constantly supplied PPE to HCW (99.4%) (Table 3).

### 3.5. Follow-Up and Clinical Outcomes

The COVID-19 medical group followed up with HCWs diagnosed with COVID-19 through phone or video calls using a standardized survey asking about the evolution of symptoms and providing tailored recommendations. In general, patients were contacted 15 times on average (IQR 10–18) from infection onset until return to the workplace. Additionally, 2279 people (87.3%) had a negative PCR test 14 days after the first positive PCR or antigen rapid test. At the end of follow-up, 63.9% of cases (1669 people) no longer had symptoms, and 37.7% continued having symptoms sporadically when they returned to work, including headache (12.6%), cough (9.6%), and anosmia (9.3%). We estimated a recovery time to return to workplaces at about 26–33 days after the diagnosis. Finally, 1880 people (72.0%) had a mild illness, 718 people had moderate illness (27.5%), three persons were hospitalized (0.11%), seven needed high flow oxygen (0.27%), one needed mechanical ventilation (0.04%), and only one worker (0.04%) died due to COVID-19.

## 4. Discussion

Globally, governments have proposed different guidelines to help return to work due to lockdown by the SARS-CoV-2 pandemic, including the early detection of outbreaks to create safer workplaces, especially for people with increased risk to for severe COVID-19 illness and people occupationally exposed, such as healthcare workers who are in closer contact with potential COVID-19 cases [1,2]. Therefore, the screening for SARS-CoV-2 infection and treatment of confirmed cases in this population group is essential, given the importance of HCWs for pandemics control [7]. This work aims to report the workflow implemented to reduce the incidence and lethality of COVID-19 in HCW in 129 healthcare facilities around Mexico.

An average incidence of 3 to 57% of SARS-CoV-2 infection among healthcare workers has been previously reported [1,23,24], with a mortality of 0.5 to 14.7% [24,25,26].

Here, we report a lower COVID-19 incidence and lethality (35.4% and 0.04%, respectively) (42% and 10.82%, respectively) [18,27] and lethality in HCWs from public institutions (0.82%) than observed in the general population [6]. Moreover, we have shown a lower hospitalization rate (0.11%), including lower respiratory insufficiency rate (0.27%) and mechanical ventilation rate (0.4%) than HCWs in public institutions (hospitalization = 4.12%, mechanical ventilation = 0.58%) [6]. Regarding occupational programs, similar results were reported (COVID-19 incidence = 10.6% with null mortality) in HCWs after implementing an occupational program in a population aged 36 years old with 21% comorbidity prevalence [12].

Different factors could explain the low lethality reported in this work in addition to the occupational health program, such as age since the majority of HCWs being under 40 years, the low prevalence of comorbidities (15.9%) not related to increased risk of having a severe COVID-19 illness; thus most of them could be considered as a low-risk population [28,29]. Furthermore, the majority of HCWs were women in whom lower mortality from COVID-19 compared to meng have been reported [30]; additionally, we home isolated people with clinical conditions that made them susceptible to develop severe COVID-19 illness, such as diabetes, obesity, hypertension, immunosuppressant diseases (Lupus, cancer or HIV) and pregnancy; together these factors along with the implementation of the occupational health program could explain the low incidence and COVID-19 lethality observed.

Moreover, to identify outbreaks and control the SARS-CoV-2 spread, the determination of the origin of contagion is relevant for the traceability of chain contagion, especially in healthcare facilities. We found that origin of contagion changes with pandemic growth in the country to be more frequently be community infections at the beginning of pandemic and then decreased, while internal infections increased during the first epidemic curve, then they tended to decrease; this might be due to the establishment of an occupational health program. Internal infections (59.1%) were more frequent in front-line HCWs, identifying co-workers and patients as contagion sources. That could explain the highest infection rate observed in first-line HCWs (65.8 per 100 HCW), supporting the occupational risk of SARS-CoV-2 infections among them [31].

Earlier reports identified the concern of health authorities about the supply of PPE to enhance the security of HCWs in the workplace [32]. Current information is inconsistent; some studies report that the null or inadequate usage of PPE is related to an increased risk of infection [33,34]. In contrast, others say report differences in using PPE and subsequently acquiring SARS-CoV-2 infections [35,36]. Nevertheless, the generalized use of face masks by the population, and not only for people occupationally exposed, is strongly recommended by different agencies, including the OMS and the CDC in the US [37,38]. Therefore, we continuously supplied PPE and provided training for all HCWs to use it correctly. Additionally, further training was delivered, such as the early recognition of symptomatic COVID-19 cases, hand washing, and surface disinfection.

Different artificial intelligence-related approaches have been tested to detect face mask use and body temperature measuring [39,40,41]. Some difficulties remain in that field, such as the detection of different mask types, different degrees of obstructions, among others [42]. However, that technology could help rapidly improve the contact-less detection of suspicious COVID-19 cases and similar respiratory diseases, such as flu.

Some limitations of this work are the bias in the sex proportions because we enrolled more women than men; however, infection rates were similar without significant statistical difference. Moreover, we included rapid antigen detection tests that could miss detecting some COVID-19 cases over the PCR test. However, we used that test in settings where there were no available PCR tests, in accordance with WHO guidance [13]. Moreover, given that the average incubation time of SARS-CoV-2 is 14 days and it may take 5–6 days to show symptoms [43], this could limit identifying the contagion source since we did not retrospectively track all contacts.

Furthermore, due to the health emergency, we do not evaluate each individual’s performance of the training provided; we only supervised the use of PPE to reduce SARS-CoV-2 incidence in our clinics; however, other studies showed a negative impact of COVID-19 in populations that did not use masks and in HCWs with inadequate or null training in the use of PPE [44,45].

Regarding strengths, the continuing supervision helped us to early-detect outbreaks and contagion chains in our clinics, identify suspected COVID-19 cases, and deliver updated training information. The follow-up COVID-19 cases also allowed us to make medical recommendations to prevent the development of severe illness and drastically reduce the mortality compared with other healthcare institutions in the country [26,31]. Finally, we consider that this work could help other workplaces to control outbreaks and create guidelines for safe back-to-work.

## 5. Conclusions

The occupational program implemented allowed us to significantly reduce the incidence (35.4%), hospitalization (0.11%), and lethality (0.04%) of COVID-19 in healthcare workers. This work could help other workplaces implement similar workflows to facilitate safe environments during the SARS-CoV-2 pandemic and in other similar contexts.

## Figures and Tables

**Table 1:** Infection rate by sex, age, and type of work.

Characteristic	Number of People	Number of COVID-19 Cases	Infection per 100 HCWs	95% CI	*p*-Value
Sex					
Female	5414	1946	35.9	34.7–37.2	0.0983
Male	1962	664	33.8	31.8–36.0	
Overall	7376	2610	35.4	34.3–36.5	-
Age (years)					
<30	3556	1570	44.2	42.5–45.8	<0.0001 *
30–39	2781	828	29.8	28.1–31.5	
40–49	760	172	22.6	19.8–25.7	
50–59	279	40	14.3	10.7–19.0	
Type of worker					
Front-line HCWs	2251	1481	65.8	63.8–67.7	<0.0001
Non-front-line HCWs	5125	1129	22.0	20.9–23.2	

Differences were tested with the Fisher exact test, and *p*-values were two-tailed. * Differences between age groups were analyzed by the Chi-test for trend (χ^2^ = 254.7, df = 1).

**Table 2:** Sociodemographic characteristics of HCWs with COVID-19.

Characteristic (*n* = 2610)	Number of People	% (95% CI)
**Age**		
Median (IQR)	28 (25–33)	---
**Sex**		
Female	1946	74.6 (72.9–76.2)
Male	664	25.4 (23.8–27.2)
**Symptoms**		
No	263	10.1 (9.0–11.3)
Yes	2347	89.9 (88.7–91.0)
Comorbidities		
No	2196	84.1 (82.7–85.5)
Yes	414	15.9 (14.5–17.3)
**Smoking** ^a^		
Non-Smokers	2208	84.6 (83.2–85.9)
Passive smokers	100	3.8 (3.2–4.6)
Smokers	300	11.5 (10.3–12.8)
**Type of Transport** ^a^		
Public	1708	65.4 (63.6–67.2)
Private	779	29.9 (28.1–31.6)
Not use	121	4.6 (3.9–5.5)

^a^ Missing data = 2.

**Table 3:** Personal protective equipment and training received by participants.

Characteristic ^a^ (*n* = 2610)	Number of People	% (95% CI)
**Use of PPE**		
No	15	0.6 (0.4–0.9)
Yes	2595	99.4 (99.1–99.7)
**PPE type** ^b^		
Polycarbonate face shield	2520	96.6 (95.8–97.2)
Surgical mask	2582	98.9 (98.5–99.3)
N95 mask	2132	81.7 (80.2–83.1)
Hand gel	1243	47.6 (45.7–49.5)
Gloves	1087	41.7 (39.8–43.6)
Disposable gown	611	23.4 (21.8–25.1)
Shoe cover	562	21.5 (20.0–23-.2)
**Training**		
No	2	0.08 (0.02–0.28)
Yes	2608	99.9 (99.7–100)
**Training type** ^c^		
Hand washing	2450	93.9 (92.9–94.7)
Work area disinfection	2465	94.4 (93.5–95.3)
Use of mask	2238	85.8 (84.4–87.0)
Use of antibacterial gel	1404	53.8 (51.9–55.7)
Use of face mask	1213	46.5 (44.6–48.4)
COVID-19 symptoms recognition	857	32.8 (31.1–34.7)
**Training media** ^d^		
Institutional website	282	10.8 (9.7–12.1)
Platform Universidad Salud Digna	1440	55.2 (53.3–57.1)
Video recording	1350	51.7 (49.8–53.6)
Video call	1093	41.9 (40.0–43.8)
Slide presentation	660	25.3 (23.7–27.0)
Institutional bulletin	405	15.5 (14.2–17.0)
Email	364	13.9 (12.7–15.3)

^a^ Missing data= 2; ^b–d^ Some categories are not exclusive.

## Data Availability

The data and dashboards are not publicly available to protect the personal data of the participants.

## Supplementary Materials

The following are available online at https://www.mdpi.com/article/10.3390/ijerph182211772/s1, Figure S1: Diagram flow for triage and screening of suspected COVID-19 cases in ambulatory clinics, Table S1: Questionnaire for detection of COVID-19 suspicious cases by EpiSD app, Table S2: Questionnaire for epidemiologic study, Figure S2: Diagram flow to discharge follow-up cases and back to work, and Figure S3: Examples in Spanish of a dashboard for analysis of epidemiological information and the follow-up cases.
