# Supplementary material for: Control and Prevention of SARS-CoV-2 Outbreaks among Healthcare Workers from 129 Healthcare Facilities in Mexico
Source: Int J Environ Res Public Health. 2021 Nov 10;18(22):11772. doi: 10.3390/ijerph182211772 (PMC8625209; doi:10.3390/ijerph182211772)
Supplement: Supplementary file 1 [file ijerph-18-11772-s001.zip › ijerph-1388989-supplementary.pdf]

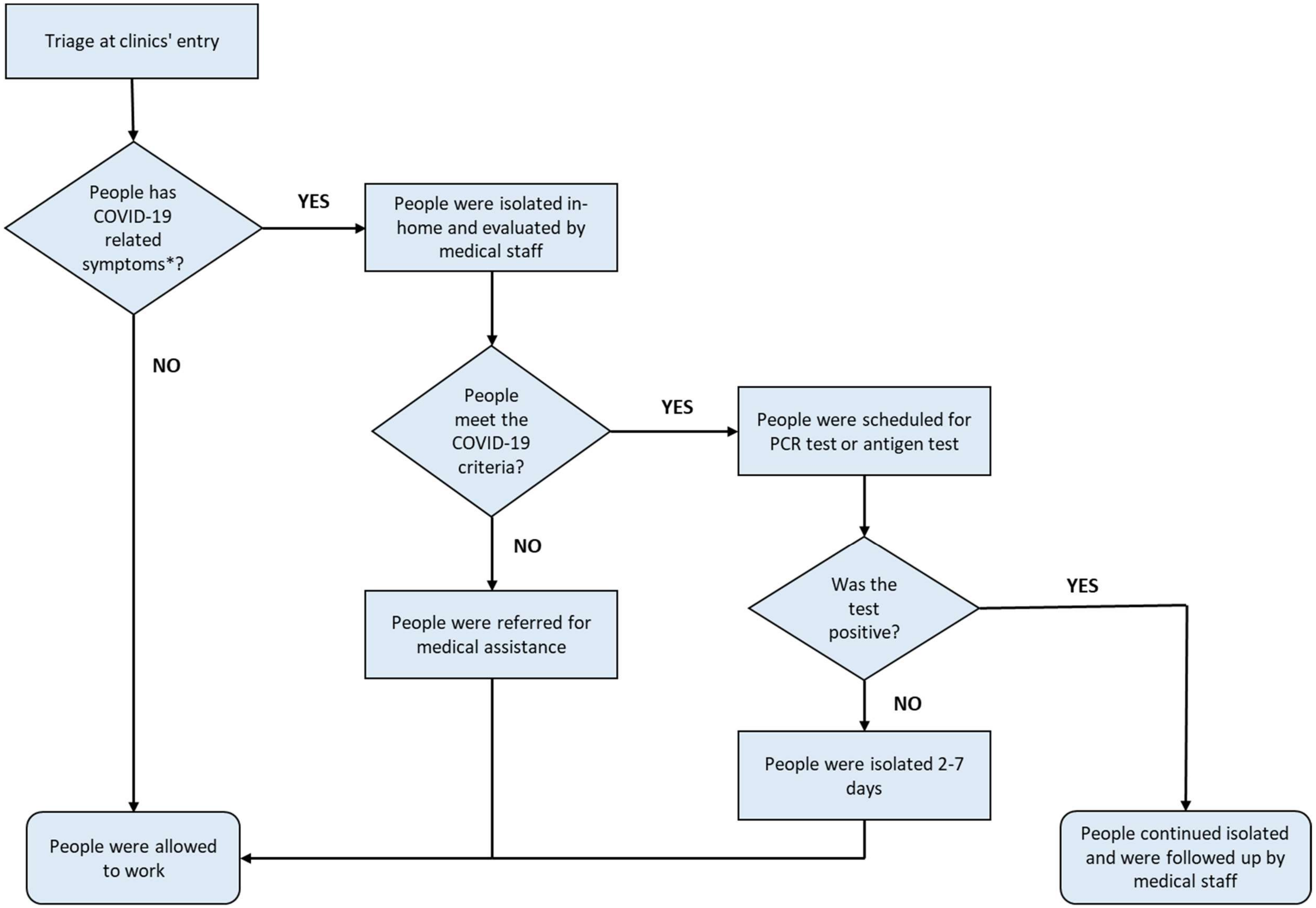

Supplementary Figure S1. Diagram flow for triage and screening of suspected COVID-19 cases in ambulatory clinics. At entry of each clinic we implemented a triage to all HCW that includes measuring body temperature, looking for fever (T ≥ 38°C), and asking for COVID-19 related symptoms (*) in the last 24 hrs. Such as fever, cough, breath difficulty, chest pain, body pain, chills, anosmia and ageusia.

Supplementary Table S1. Questionnaire for detection of COVID-19 suspicious cases by EpiSD app

Triage by EpiSD tool from COVID-19 control program
1. Name<br>2. Registration date<br>3. Birth date<br>4. Clinic's affiliation<br>5. Job<br>6. Phone<br>7. Have you been in touch with someone confirmed with COVID-19?<br>8. Have you been in touch with someone suspected of COVID-19?<br>9. Have you had a fever greater than 38 degrees?<br>10. Have you had a constant, dry cough?<br>11. Have you had trouble breathing?<br>12. Have you had a loss of smell?<br>13. Have you had a loss of taste?<br>14. Have you had a headache?<br>15. Have you had a runny nose?<br>16. Have you had muscular pain?<br>17. Have you had a loss of taste?<br>18. Have you had conjunctivitis?<br>19. Have you had pain when swallowing?<br>20. Have you had diahrrea?<br>**21.** What was the date of onset of symptoms?

Supplementary Table S2. Questionnaire for epidemiologic study

Epidemiological information of COVID-19 positive cases
1. Case number<br>2. Sex<br>3. Age<br>4. Birth date<br>5. State of Residence<br>6. Clinic's affiliation<br>7. Job<br>8. Have you previously received the influenza vaccine?<br>9. Are you pregnant of breastfeeding?<br>10. What was the date of onset of symptoms?<br>11. What was the sampling date?<br>12. Do you receive PCR or rapid antigen test?<br>13. How many tests have you received?<br>14. Have you been in touch with someone confirmed with COVID-19?<br>**15. Have you had any of the following comorbidities?**<br>Hypertension<br>Diabetes<br>Obesity<br>Hyper/<br>Hypothyroidism<br>Asthma<br>Arthritis<br>Cardiac disease

Cancer
Renal insufficiency
HIV
Pulmonary hypertension
COPD
Lupus
Hepatic insufficiency
Smoking

16. Have you had any of the following symptoms?

Headache
Myalgia or Arthralgia
Sore throat
Cough
Runny nose
Fever
Chills
Diarrhea
Breath difficulty/Chest pain
Chest pain
Abdominal pain
Conjunctivitis
Anosmia
Ageusia
Vomit

17. Wich of the following protective equipment (PPE) you use?

Face mask
Mask
N95
Hand gel
Gloves
Disposable gown
Shoe cover

18. Wich of the following training programs did you receive?

Hand washing
Work area disinfection
Use of mask
Use of antibacterial gel
Use of face mask
COVID-19 symptoms recognition

19. Through what media did you receive your training?

Institutional website
Platform Universidad Salud Digna
Video recording
Videocall
Slide presentation
Institutional statement
Email

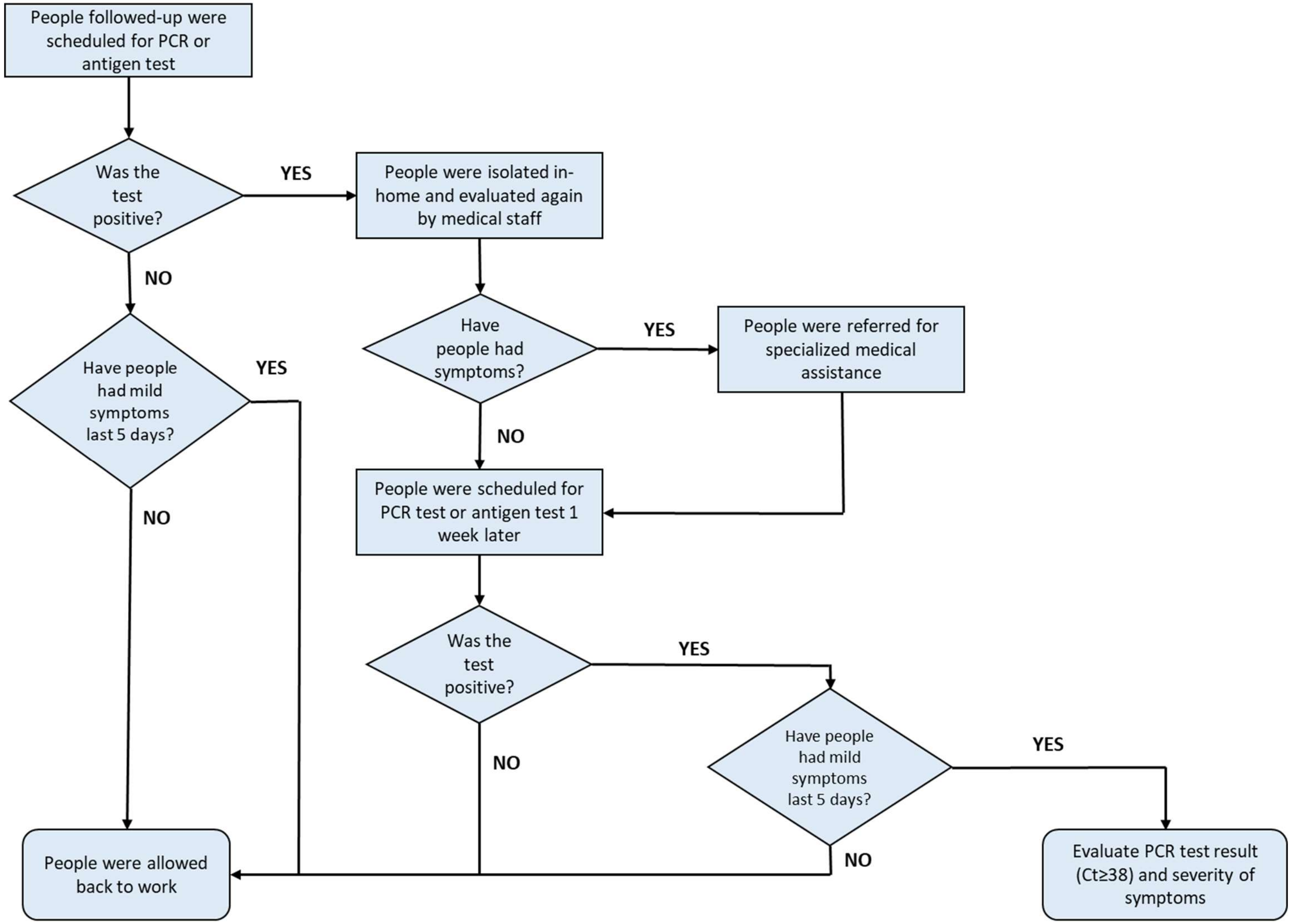

Supplementary Figure S2. Diagram flow to discharge follow-up cases and back to work.
COVID-19 cases were followed-up till 14 days after symptoms onset, then were scheduled for PCR test or rapid antigen test. We used a combination of test results and the presence of mild symptoms to make discharge criteria.

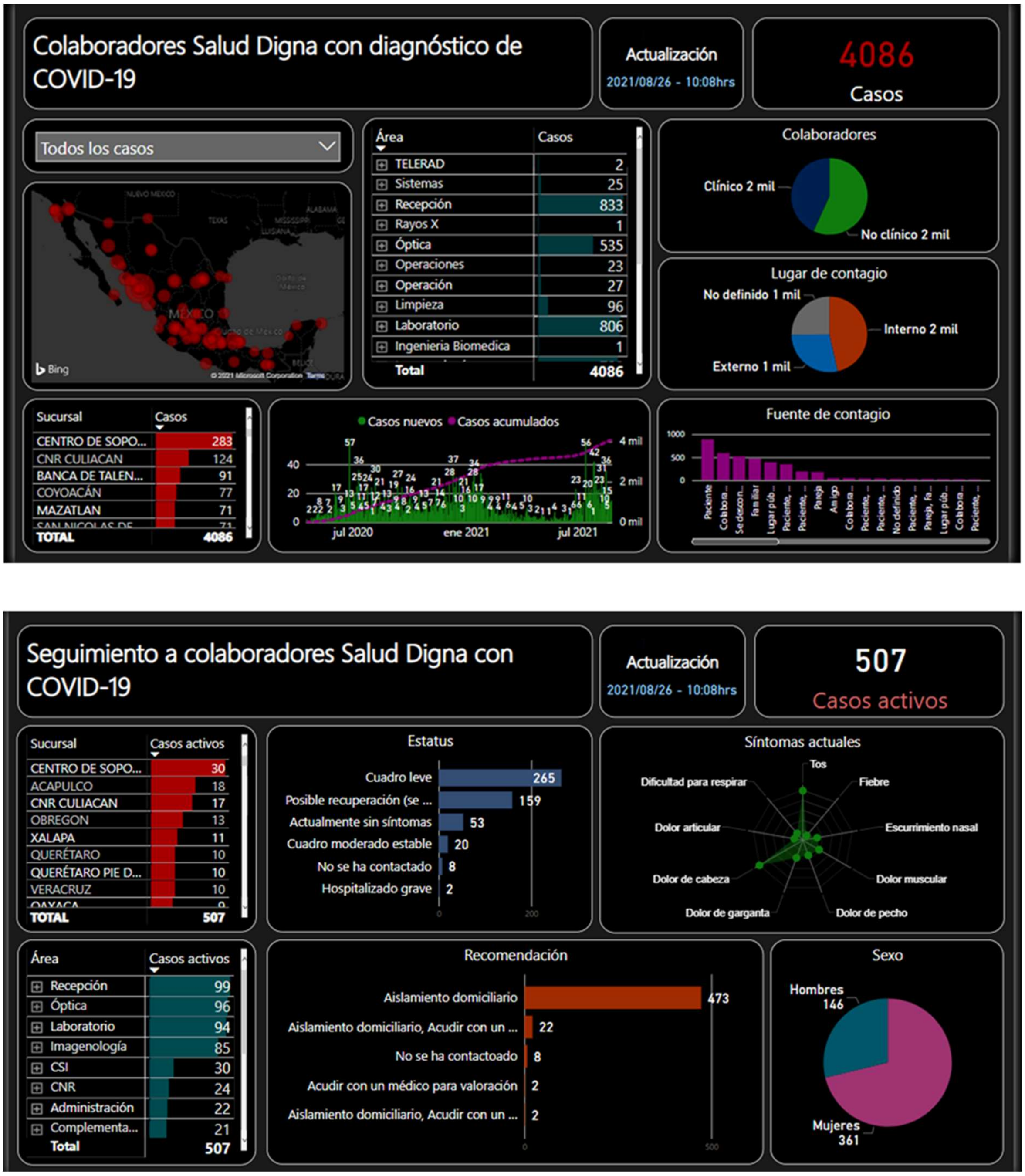

Supplementary Figure S3. Examples in Spanish of a dashboard for analysis of epidemiological information and the follow-up cases. These examples correspond to a dashboard from general information showing: the total of cases registered, daily incidence, the geographic distribution, type of job (first-line HCW and non-fisrt-line HCW) and source of contagion (upper panel); Clinic, status of cases, symptoms, sex, and medical opinion (lower panel) (Dashboard retrieved 30th August, 2021).
